# Comparative evaluation of gingival displacement and clinical efficacy using polyvinyl siloxane foam and retraction cord systems: A randomized controlled in vivo study

**DOI:** 10.1016/j.jobcr.2025.07.016

**Published:** 2025-07-26

**Authors:** Aditya Acharya, K.P. Lekha, Raisa Chodankar, Yash Alpesh Zawar, Konark Patil, Adithi Rao

**Affiliations:** a Department of Prosthodontics, KLE Academy of Higher Education and Research, Deemed-to-be-University, Belagavi, Karnataka, India; b Department of Prosthodontics, SDM College and Hospital, Dharwad, Karnataka, India; c Department of Orthodontics and Dentofacial Orthopedics, KLE Academy of Higher Education and Research, Deemed-to-be-University, Belagavi, Karnataka, India; d Department of Prosthodontics, Al Badar Rural Dental College & Hospital, Gulbarga, Karnataka, India

**Keywords:** Gingival displacement, Gingival retraction, Magic foam cord, Retraction cord

## Abstract

Every tooth in the arch and the soft tissues around the prepared tooth need to be replicated in the impression. To prevent tearing during impression removal, it is essential to ensure sufficient sulcus width. To date, mechanical, chemico-mechanical, electrosurgical, surgical, and laser methods have been used to accomplish gingival retraction. The purpose of both clinical and laboratory analysis of the efficacies of chemically impregnated retraction cord and polyvinyl siloxane foam retraction systems is based on the relative amount of vertical and horizontal gingival displacement, time of placement, and the presence or absence of bleeding.

**Methods and materials:**

A total of 30 participants aged 20–40 years were enrolled in a randomized controlled trial and quality assessment was conducted according to the CONSORT checklist (CTRI/2022/10/046181). In a split-mouth design, retraction was done using 25 % aluminium sulfate-impregnated retraction cords and Magic FoamCord (MFC). The Mann-Whitney and T-tests were used for data analysis.

**Result:**

Mann-Whitney Test concluded that for vertical gingival retraction cord and Magic foam at 2nd M are statistically insignificant in all three sites (p > 0.05). The mean horizontal displacement achieved at the second molar and second premolar for retraction cord was 0.36 ± 0.07 mm, which was greater than MFC, 0.24 ± 0.06 mm (p = 0.001; 95 % CI). The T-test used for the time of placement between retraction cord and magic foam cord was significant (p < 0.001). The gingiva was observed for presence or absence of bleeding soon after retrieval of the retraction cord and the MFC.

**Conclusion:**

Retraction cords provide greater horizontal displacement but are more time-consuming and traumatic compared to MFC, which is more time-efficient and less invasive. This highlights the need to balance efficacy and efficiency in clinical practice.

## 1 Introduction

The success and longevity of fixed dental restorations depend heavily on the health and stability of the supporting periodontium.^1^ Achieving this stability is particularly challenging when dealing with subgingival margin preparation, which is often necessitated by factors such as caries, existing restorations, cosmetic considerations, or the need for enhanced retention.^2^ Accurate replication of the prepared tooth and surrounding soft tissues is crucial for ensuring the long-term success of the restoration. This requires an impression that can capture fine marginal details without distortion or tearing during removal.^2^

In addition, fixed prostheses with subgingival margins have been shown to influence gingival health, requiring careful consideration of margin placement and retraction techniques.^30^ To accomplish this, the gingival sulcus must be sufficiently displaced laterally and vertically to accommodate the low-viscosity impression material.^3^ This process, known as gingival displacement, creates a defined space between the prepared finish line and the gingiva, facilitating the precise application of impression material. Various methods, including mechanical, chemico-mechanical, electrosurgical, and laser techniques, have been employed to achieve gingival retraction.^3^, ^4^, ^5^, ^6^

Retraction cords remain one of the most established and widely used techniques due to their predictability, safety, and effectiveness compared to methods like gingival curettage and electrosurgery.^7^ However, the development of innovative materials like polyvinyl siloxane (PVS) foams has provided clinicians with alternatives that aim to improve patient comfort and procedural efficiency. Magic FoamCord, a pioneering PVS-based material, represents a significant advancement in gingival displacement techniques. During its setting reaction, hydrogen gas is released, creating a sponge-like structure that gently expands the sulcus. A comprecap is then used to apply pressure, enhancing the material's retraction capabilities.^7^^,^^8^, ^9^

Despite advancements in retraction materials and techniques, there remains a lack of consensus regarding the most effective approach. The absence of standardized evaluation criteria complicates the comparison of horizontal and vertical gingival displacement achieved by different methods. This study was designed to analyze and compare the performance of chemically impregnated retraction cords and PVS foam systems in terms of gingival displacement, placement time, and bleeding.

Null Hypothesis: There is no significant difference in gingival displacement, time efficiency, or bleeding between retraction cord and polyvinyl siloxane foam systems.

## 2 Materials and method

### 2.1 Study design, setting, and ethical considerations and trial registration

A randomized controlled trial was conducted at the Department of Prosthodontics, Crown and Bridge from October 2022 to July 2023. The study was approved by the institutional ethics committee (Ref ICE/KLE VKIDS/2022/48) and registered with the Clinical Trial Registry of India (CTRI/2022/10/046181). This study was conducted as a pilot trial with a convenience sample size of 30 sites, based on feasibility and available resources. A formal power analysis was not performed due to limited prior clinical data using the specific foam-based system evaluated. All participants provided written informed consent. The study was assessed using the CONSORT checklist.^10^

### 2.2 Eligibility criteria for participants, sampling, and randomization

Inclusion criteria: Participants aged 20–40 years requiring mandibular first molar replacement, with clinically healthy gingiva (assessed using the Loe and Silness Gingival Index), radiographically sound abutments, and willingness for full-coverage restoration.

Exclusion criteria: Tipped, rotated, or anomalous abutments; gingival or periodontal disease; systemic health issues; or regressive age-related changes.

**Fig. 1:**
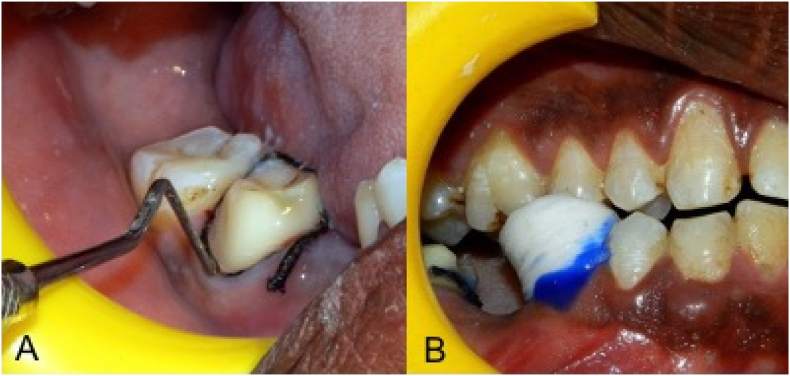
(A) Gingival retraction using a retraction cord impregnated with 25 % aluminium sulfate gel; (B) Polyvinyl siloxane foam with an anatomic comprecap.

Fifteen participants were assigned to receive gingival retraction using a retraction cord (#00 Ultrapak, Ultradent Inc., Utah, USA) impregnated with 25 % aluminium sulfate gel (Gel Cord, Pascal International Inc., Washington, USA) on the second molar and polyvinyl siloxane foam (Magic FoamCord, Coltene/Whaledent Inc., Altstätten, Switzerland) with an anatomic comprecap on the second premolar (Fig. 1). In the next fifteen participants, the placement was reversed. Randomization was done using a computer-generated sequence.

### 2.3 Clinical procedure and parameter analysis

**Fig. 2:**
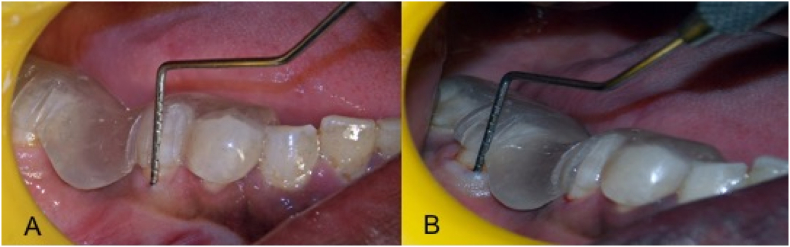
Customized oral stent.

Preliminary elastomeric impressions (Aquasil soft putty- DENTSPLY Germany, Reprosil PVS light DENTSPLY caulk- USA) were made of the mandibular arch using metallic dentulous perforated stock tray, which served as a control for comparison of gingival displacement after tooth preparation. Using Self cure clear acrylic resin (DPI Mumbai), a customized oral stent of the individual was fabricated extending from the first mandibular premolar to the retromolar area. The stent had three indentations each for the calibrated probe on the second premolar and the first molar corresponding to the mesio-buccal, mid-buccal and disto-buccal regions. Abutments were prepared for full coverage restoration following all the principles of tooth preparation given by Shillingburg et al.^11^ shoulder finish line margins of width 1.2–1.5 mm were placed on the buccal aspect of the teeth and chamfer finish line margins were placed on the lingual aspect. The customized oral stent was positioned and the sulcus depth was measured at the mesio-buccal, mid-buccal, and disto-buccal regions on both abutment teeth before any retraction techniques were applied (Fig. 2). The two gingival retraction systems were used on the prepared abutment teeth according to the manufacturers recommendation. After subjecting the patients to the retraction procedures, and recording of vertical displacement, a final impression was made using elastomeric impressions (Aquasil soft putty- DENTSPLY Germany, Reprosil PVS light DENTSPLY caulk- USA) impression was retrieved and inspected for voids and any other irregularities and subjected to microscopic analysis. If any shortcomings were found in the obtained impression, the entire procedure from retraction to impression making was repeated.

**Fig. 3:**
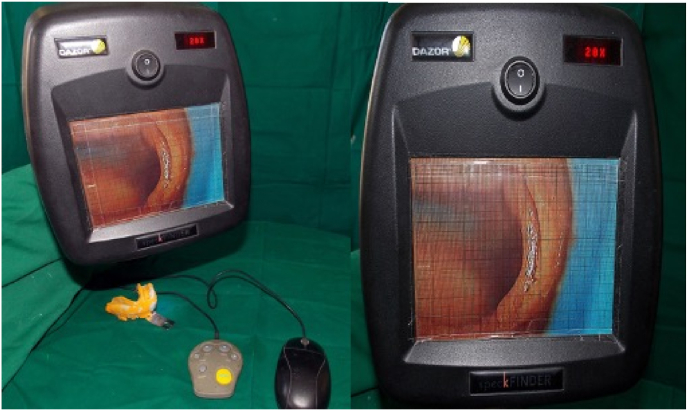
Microscopical evaluation of the pre- and post-gingival retraction elastomeric impressions done using the SPECK FINDER^R^ at a magnification of 20×.
• **Time taken for cord placement:** Post tooth preparation of the abutments the application time of the two retraction systems in both situations was timed using a stopwatch.
• **Presence of bleeding**: Upon retrieval of the retraction cord and the MFC, the gingival sulcus was noted for the presence or absence of active bleeding.

• **For vertical displacement:** The graduated William's probe (Hu Freidy –USA) was again used along the customized oral stent, and recordings were made in the three different locations along the buccal margins at the mesio-buccal, mid-buccal and disto-buccal areas and the measurements were noted.
• **For horizontal displacement**: Microscopical evaluation of the pre- and post-gingival retraction elastomeric impressions was done using the SPECK FINDER^R^ at a magnification of 20× (Fig. 3). A customized measurement chart with the smallest measurement of 0.1 × 0.1 mm was made and put up on the display screen. The amount of gingival retraction was calculated by taking the difference between the values attained before and after gingival retraction.

### 2.4 Statistical analysis

Data entry and analysis were performed using Microsoft Excel and SPSS v22 (IBM, USA). First, the Shapiro–Wilk test evaluated normality of paired differences. Variables meeting normality assumptions (e.g., vertical displacement and placement time) were analyzed with paired two-tailed t-tests, while non-normal variables (e.g., horizontal displacement) were compared using two-tailed Wilcoxon signed-rank tests, the nonparametric equivalent. All hypothesis tests were two-tailed to detect differences in either direction, with a significance threshold of p < 0.05. Results are presented as mean ± SD or median (interquartile range) as appropriate, with 95 % confidence intervals for effect estimates. Effect sizes (Cohen's d for parametric tests; rank-biserial correlation for nonparametric tests) were calculated for primary outcomes to quantify the magnitude of observed differences.

## 3 Results

30 participants in total, 16 men and 14 women met the requirements for inclusion. A total of 60 sites were assessed of which 30 were second molar and 30 s premolar. At the time of the study, a mean age of 29.63 ± 4.80 years was analyzed.

The second molar and second premolar's mean and standard deviation of the vertical gingival retraction achieved with retraction cord and magic foam are stated in Table 1. Mann-Whitney Test concluded that retraction cord and Magic foam at 2 nd M are statistically insignificant in all three sites (p = 0.05). Retraction cord and Magic foam at 2nd premolar are statistically insignificant in all three sites (p > 0.05).

The mean horizontal displacement achieved at the second molar for retraction cord was 0.36 mm and that of MFC was 0.24 mm with standard deviation value of 0.07 mm. The mean horizontal displacement achieved at the second premolar for retraction cord was 0.33 mm with standard deviation value of 0.04 mm and that of MFC was 0.22 mm with standard deviation value of 0.03 mm **(**Table 2). Mann-Whitney Test and it was seen that in case of the second molar and second premolar the retraction cord and MFC group differ significantly [p = 0.001].

**Table 1:** Descriptive statistics for vertical gingival depth (in mm).

LOCATION	RETRACTION SYSTEMS	VERTICAL GINGIVAL DEPTH (IN MILLIMETERS)			
			MESIO-BUCCAL	MID-BUCCAL	DISTO-BUCCAL
AT SECOND MOLAR	RETRACTION CORD	MEAN	0.85	0.55	0.43
		SD	0.06	0.03	0.05
	MFC	MEAN	0.66	0.37	0.32
		SD	0.05	0.03	0.07
AT SECOND PREMOLAR	RETRACTION CORD	MEAN	0.73	0.56	0.44
		SD	0.06	0.02	0.03
	MFC	MEAN	0.56	0.47	0.31
		SD	0.04	0.02	0.05

SD: Standard deviation.

**Table 2:** Descriptive statistics for Horizontal displacement (in mm) SD: Standard deviation.

Groups	RETRACTION CORD 1	MFC 1	RETRACTION CORD 2	MFC 2
**Mean**	0.36	0.22	0.33	0.24
**SD**	0.08	0.03	0.04	0.07

For the average amount of time needed to position the two-retraction systems T-test was used; As the p-value <0.001 the time of placement between retraction cord and magic foam cord was significant (Table 3).

**Table 3:** Time taken for placement (in seconds).

Groups	RETRACTION CORD 1	MFC 1	RETRACTION CORD 2	MFC 2
**Mean**	s	47.93	147.40	69.00
**SD**	15.84	5.26	9.80	5.42

The gingiva was observed for presence or absence of bleeding soon after retrieval of the retraction cord and the MFC. It was found that in 11 cases there was presence of active bleeding when retraction cord was used in a molar and only in 4 cases with that of magic foam cord. Whereas in case of second premolar, there was no bleeding seen with magic foam cord, but of active bleeding was noted in 11 subjects.

## 4 Discussion

Accurate reproduction of the finish line during impression-making is essential to ensure the success of restorations. Failure to achieve this can result in marginal discrepancies that lead to recurrent caries, gingival irritation, or periodontal damage. Effective gingival retraction is critical for producing error-free impressions. While newer pastes and gels have gained popularity, retraction cords remain the standard due to their reliability and effectiveness.^12^

Retraction cords offer predictable results but come with challenges such as prolonged application time, potential discomfort, and risks of gingival trauma or recession if improperly handled.^12^^,^^13^ Nonmedicated cords are safe but lack effective hemorrhage control, leading to the development of medicated cords. Studies indicate that buffered aluminum sulfate (55 %) is the most commonly used medicament with retraction cords, followed by ferric aluminum chloride and ferric sulfate (23 %).^14^

Alternative techniques, such as polymer-based cords and gingival retraction pastes, provide additional options. Merocel, a hydroxylated polyvinyl acetate material, is highly effective in absorbing intraoral fluids and achieving sulcus displacement.^14^ Similarly, Expasyl paste combines kaolin for gingival displacement and 15 % aluminum chloride for hemostasis, offering a painless and quick application, though it is less effective for subgingival margins.^16^ Magic FoamCord (MFC), a PVS material, eliminates the need for cord packing by expanding gently within the sulcus during its setting reaction. This method has been associated with lower levels of inflammatory cytokines in gingival crevicular fluid compared to traditional cords.^15^, ^17^, ^18^

Our study found that retraction cords achieved greater gingival displacement but were associated with bleeding and required significantly more time than MFC^19^, ^20^. This aligns with previous research by Kumari S et al., which reported that cord placement required four times the duration of MFC^8^, ^24^, ^25^. While Singh AA observed a slight, statistically insignificant advantage in displacement with cords (0.8 % greater than MFC), both methods exceeded the minimum sulcus width reported in the literature^21^, ^22^, ^23^. Mehta S et al. also reported significant differences in sulcus width among these methods.^7^ Studies by Madaan et al.^27^ and Mathew et al.^28^ confirm that Magic FoamCord offers a less traumatic alternative. Additionally, Ünalan Değirmenci et al.^29^ emphasized the importance of evaluating periodontal health after retraction, with findings indicating that PVS-based systems promote better healing.

Gingival zenith and esthetics must also be preserved in subgingival preparations. Retraction-induced trauma can compromise tissue stability. Foam-based systems may offer a balance between effectiveness and soft tissue preservation.^3^, ^26^

A strength of this study is its randomized split-mouth design, minimizing intersubject variability. Additionally, the use of customized stents ensured precise and repeatable measurements. The study's limitations include its small sample size and focus on mandibular premolars and molars. Future research should include a broader range of participant ages, gingival biotypes, and additional tooth types, as well as a histological analysis of retraction materials' effects on soft tissues.

Success in gingival retraction ultimately depends on the clinician's ability to select the most appropriate technique based on case-specific factors such as tooth position, gingival tissue condition, and impression material.^12^

## 5 Conclusion

Both systems achieved clinically acceptable vertical sulcus openings—there was no statistically significant difference in vertical displacement (p > 0.05). Retraction cords produced greater horizontal displacement (0.36 mm vs. 0.24 mm; p = 0.001) but required significantly longer placement times (p < 0.001) and were associated with more bleeding (p = 0.04). Conversely, Magic FoamCord offered advantages in ease of use, reduced trauma, and shorter clinical application time.

Clinicians should weigh the specific demands of each case—such as the need for greater displacement versus minimal invasiveness—when selecting a retraction system. In modern restorative practices, especially where patient comfort and digital workflows are prioritized, foam-based systems like Magic FoamCord present a viable alternative to conventional cords.

## Sources of funding declaration

No funding sources were involved in the preparation of this manuscript.

## Declaration of competing interest

The authors declare that they have no known competing financial interests or personal relationships that could have appeared to influence the work reported in this paper.
